# International perspectives on physician knowledge, attitudes, and practices related to medical cannabis

**DOI:** 10.3389/fpubh.2025.1463871

**Published:** 2025-02-20

**Authors:** Shariful A. Syed, Jatinder Singh, Hussien Elkholy, Irena Rojnić Palavra, Marko Tomicevic, Anamarija Petek Eric, Mariana Pinto da Costa, Sinan Guloksuz, Rajiv Radhakrishnan

**Affiliations:** ^1^Department of Psychiatry, Yale School of Medicine, New Haven, CT, United States; ^2^Department of Psychiatry, Penn State Milton S. Hershey Medical Center, Hershey, PA, United States; ^3^Neurology and Psychiatry Department, Faculty of Medicine, Ain Shams University, Cairo, Egypt; ^4^University Psychiatric Hospital Sveti Ivan, Zagreb, Croatia; ^5^Department of Psychiatry, University Hospital Dubrava, Zagreb, Croatia; ^6^Department of Psychiatry, Clinical Hospital Centre, Osijek, Croatia; ^7^Faculty of Medicine Osijek, J.J. Strossmayer University, Osijek, Croatia; ^8^Faculty of Dental Medicine and Health, J.J. Strossmayer University Osijek, Osijek, Croatia; ^9^Institute of Psychiatry, Psychology & Neuroscience, King's College London, London, United Kingdom; ^10^Institute of Biomedical Sciences Abel Salazar, University of Porto, Porto, Portugal; ^11^Department of Psychiatry and Neuropsychology, School for Mental Health and Neuroscience, Maastricht University Medical Center, Maastricht, Netherlands

**Keywords:** medical cannabis, physician knowledge, physician practice, physician attitude, cannabis psychosis, cannabis addiction

## Abstract

**Background:**

The trends of recreational use of cannabis and the use of cannabis for medical indications (i.e., “medical cannabis”) have grown in recent years. Despite that, there is still limited scientific evidence to guide clinical decision-making, and the strength of evidence for the medical use of cannabis is currently considered to be low. In contrast, there is growing evidence of negative health outcomes related to the use of cannabis. In this rapidly shifting landscape, the role of physician attitudes regarding the therapeutic value of cannabis has become essential. This study aimed to characterize knowledge/experience, attitudes, and potential predictors of clinical practice regarding medical cannabis.

**Methods:**

We conducted a cross-sectional survey of physicians from 17 countries between 2016 and 2018. The survey consisted of questions designed to explore physician knowledge, attitude, and practices regarding the use of medical cannabis. Descriptive statistics were used to examine willingness to recommend medical cannabis for medical and psychiatric indications, followed by regression analysis to identify the predictors of physician willingness to recommend medical cannabis.

**Results:**

A total of 323 physicians responded to the survey, among which 53% were women. The mean age was 35.4 ± 9.5 years, with 10.04 ± 8.6 years of clinical experience. Clinical experience with medical cannabis was overall limited (51.4% noted never having recommended medical cannabis and 33% noted inadequate knowledge regarding medical cannabis). The majority of respondents (84%) recognized the risk of psychosis with cannabis use, while only 23% correctly identified the risk of addiction with daily cannabis use. Overall, willingness to recommend medical cannabis was the highest for chemotherapy-induced nausea (67%), refractory chronic neuropathic pain (52%), and spasticity in amyotrophic lateral sclerosis (ALS; 51%).

**Conclusion:**

This international study examining physician knowledge, attitudes, and practices related to medical cannabis revealed that there are significant gaps in domain-specific knowledge related to medical cannabis. There is a wide variability in willingness to recommend medical cannabis, which is not consistent with the current strength of evidence. This study thus highlights the need for greater education related to domain-specific knowledge about medical cannabis.

## Introduction

The US government first began regulating cannabis use in 1937, and since then, the medical utility of cannabis has been open to debate (1). In 1970, cannabis acquired Schedule I drug designation under the Controlled Substances Act (2)—a designation indicating an absence of medical value and a high potential for abuse. Similar legal restrictions throughout the world have limited the overall accessibility and availability of cannabis for all uses (3). As of March 2023, medical cannabis has been approved in 39 states (including the District of Columbia, Guam, and Puerto Rico) in the United States and 41 countries worldwide (3).

Medical cannabis refers to the use of cannabis, including its constituents (i.e., delta-9 tetrahydrocannabinol (THC), cannabidiol (CBD), and other cannabinoids), as a physician-recommended treatment. Cannabis contains more than 450 chemical compounds (4), of which approximately 100 are cannabinoids (5–8). There is fair evidence supporting the use of medical cannabis in the treatment of chemotherapy-induced nausea and vomiting, anorexia associated with weight loss in AIDS patients, neuropathic pain, and spasticity in multiple sclerosis (9, 10). The evidence to support its use for Crohn’s disease, hepatitis C, Parkinson’s disease, Tourette syndrome, and glaucoma is limited (9, 11–13). In contrast, current evidence also supports the association of cannabis use with the onset and worsening of psychiatric disorders (14–17).

Approximately 2 million individuals in the United States utilized medical cannabis in 2019 through state-licensed dispensaries or home cultivation (18). In this rapidly shifting landscape of medical cannabis over the last decade, the role of physician knowledge, attitudes, and practices has become essential given that it is touted as a bona fide medical treatment.

Physicians have a duty to act in the best interest of the patient and to serve as a medical expert who can guide patients in making medical decisions while balancing the risks vs. benefits of a particular treatment modality (19). Little is known of physician knowledge, attitudes, and practices regarding medical cannabis. Pre-existing beliefs and attitudes toward cannabis are likely to influence the prescribing practice of physicians (20).

In addition to the lack of robust evidence for medical use of cannabis, there are a number of factors that impact physician attitudes. These include lack of consistency in the list of approved indications across states and countries (21, 22), significant variability in chemical constituents (components, purity, and contaminants) of medical cannabis, and discrepancy regarding its legal status at the federal and state levels (12, 23–26). In this dynamically shifting landscape, the role of physician knowledge, attitudes, and practices related to medical cannabis is important. Previous studies have shown that physicians rarely discuss the role of medical cannabis with patients (27). There is also evidence that clinical training regarding medical cannabis is limited in medical schools (28). This study aimed to characterize the knowledge, attitudes, and practices of physicians regarding medical cannabis.

## Methods

### Materials

We developed a questionnaire to assess the current knowledge, attitudes, and practice toward the medical use of cannabis (included in Supplementary material). Responses were provided in the form of multiple choice questions, numeric sliding scales, and open-ended questions. The questionnaire consisted of the following main sections: demographics, clinical practice characteristics, disorder-specific treatment efficacy, perceived proficiency in prescribing medical cannabis, risks associated with medical cannabis, and personal belief/preference for medical cannabis. Attitudes toward medical cannabis were assessed using case vignettes of different disorders, and physicians’ willingness to recommend medical cannabis was measured on a Likert scale of 0–100 (0 = not willing, 50 = equally willing/unwilling, and 100 = very willing). Belief regarding the utility of medical cannabis was assessed using the following question, which was scored as “Yes” or “No”: Hypothetically, if you had a condition that qualified for medical cannabis would you opt to get a prescription for yourself?

### Data collection

Study data were collected and managed using QUALTRICS hosted at Yale University. QUALTRICS is a secure, web-based application designed to support data capture for research studies. Data were collected between 1 March 2014, and 30 May 2018, using convenience sampling. Online Qualtrics survey was emailed to physicians through members of the World Psychiatric Association (WPA) Early Career Section. This study was reviewed and approved by the Yale Institutional Review Board.

### Analysis

All data analyses were completed using SPSS version 28. Descriptive statistics were used to summarize the characteristics of physician respondents, which were divided into various categories, including physician characteristics, medical training, clinical experience/practice characteristics, knowledge of medical cannabis, and perceived competence in relation to medical cannabis (including prescribing behavior and personal beliefs). To explore physician knowledge characteristics and individual beliefs toward medical cannabis, we classified respondents as those who reported ‘not knowing enough’ vs. ‘never recommend medical cannabis but open to it’. Student’s *t*-test was used to compare the differences in belief about the utility of cannabis use. Data plots were visualized to ensure that outlier-driven relationships did not confound the findings.

## Results

A total of 323 physicians responded to the survey, among which 53% were women. The mean age was 35.4 ± 9.5 years, with 10.0 ± 8.6 years of clinical experience. The demographics of the participants, their practice settings, self-reporting proficiency, experience with cannabis prescription, and characteristics of patient population composition are detailed in Table 1. Responses were received from physicians across 17 countries, namely, Australia (3), Canada (1), Croatia (70), Egypt (50), El Salvador (1), India (31), Indonesia (5), Peru (2), Poland (23), Portugal (62), Qatar (1), Russia (1), Saudi Arabia (2), Spain (1), South Africa (1), Turkey (62), and the United States (36).

**Table 1:** Characteristics of physician respondents.

Sex	Female	53%		
	Male	47%		
Age	35 ± 9.5 years			
Years in practice	10 ± 8.6 years			
Specialty/Subspecialty training	Psychiatry	64%	Pain medicine	0.36%
	Other	17%	Palliative care	1.1%
	Family medicine	10%	Hematology/oncology	1.8%
	Internal medicine	5.6%	Infectious diseases	2.1%
	Neurology	4.6%	Addiction	11%
	Obstetrics and gynecology	2.5%	Other	32%
	General surgery	2.2%	No sub-specialty training	47%
Practice setting	Medical School/University	52%	Inpatient	48%
	Other	34%	Outpatient	52%
	Individual private practice	6.9%		
	Group private practice	3.8%		
	Medical home	1.9%		
	Veterans Affairs facility	1.2%		
Patient population treated	Child and adolescent	26%		
	Adult	68%		
	Geriatric	28%		
Experience prescribing medical cannabis	Recommend	0.71%		
	Refer out	2.5%		
	Do not recommend	12.1%		
	Do not know enough	33.2%		
	Never recommended but open	51.4%		

### Physician knowledge regarding medical cannabis

In our sample, the majority of the physicians had minimal experience with medical cannabis: 58% of responders reported never prescribing medical cannabis but were open to it, and 34% of respondents responded, ‘I do not know enough’ but were open to it. The second most common response was ‘do not know enough’. Only 3.2% of the sample reported to have experience prescribing/recommending medical cannabis (see Table 2).

**Table 2:** Willingness to treat with medical cannabis.

Case vignette/diagnosis	Mean percent
Severe nausea/vomiting in chemotherapy	67.3
ALS with severe spasticity	51.9
HIV/AIDS, low weight/appetite	51.2
Severe remitting–relapsing MS with recent optic neuritis	45.6
Alzheimer’s/Lewy body dementia	41.7
Epilepsy, refractory	38.7
Moderate to severe Parkinson’s disease	38.6
Refractory Crohn’s disease	37.0
Refractory glaucoma	35.7
PTSD	28.6
Uncontrolled tic disorder	25.7
Psoriasis without arthritic changes	23.9
Opioid abuse with anxiety	23.2
Sickle cell disease	22.4
Autism	22.1
Alcohol use, hx of hep C, recent cirrhosis	18.9
Insomnia, no medical hx	16.1

Physician knowledge regarding the addictive potential of daily cannabis use was limited. While the majority of our sample (45%) noted 10–15% chances of addiction with daily cannabis use, 23% noted a 25–50% risk of addiction more in line with the risk noted in epidemiological studies of 30% (Table 2). The majority of respondents (84%) recognized the risk between cannabis use and psychosis (Table 3).

**Table 3:** Questions pertaining to perceived adverse effects of medical cannabis.

Probability of addiction after daily use	% of respondents
<1%	19
10–15%	45
25–50%	23
>50%	13
Association between cannabis and psychosis	% of respondents
No, I do not believe so/never seen this clinically.	9.0
No, link is an artifact	7.0
Yes, I have seen cases exemplifying the association	84
Belief about the utility of medical cannabis (i.e., opting to obtain a prescription for self for a qualified condition)	% of respondents
No	47
Yes	53

### Physician attitudes toward recommending medical cannabis

Physician willingness to recommend medical cannabis was assessed using clinical case vignettes. The highest willingness to recommend medical cannabis was for patients with chemotherapy-induced side effects (67%), refractory neuropathic pain in diabetes mellitus (52%), and severe spasticity secondary to amyotrophic lateral sclerosis (ALS; 51%). The lowest probability to recommend was for patients with an isolated complaint of insomnia (16%), alcohol use disorder with liver cirrhosis (19%), and patients with autism and self-injurious behavior (22%; Table 2). The majority of physicians (53%) noted a willingness to use medical cannabis themselves for a qualifying medical condition.

## Discussion

The aim of this study was to better characterize physician knowledge, attitudes, and practice behaviors regarding medical cannabis and to elucidate potential factors influencing practice. The results are in line with the idea that scientifically validated knowledge of medical cannabis is lacking. This international cohort of survey responders suggests that overall, physicians are unclear of the potential utility of medical cannabis apart from the conditions related to cancer or terminal illnesses. Furthermore, the fact that the addictive potential of cannabis is not well understood speaks to the insufficient dissemination of the current state of knowledge. That one in five physicians significantly underestimated the addictive potential of daily cannabis use is a cause for concern, as was highlighted by subsequent findings.

These findings are consistent with other studies examining physician knowledge related to medical cannabis, which consistently show that there is a significant gap in physician knowledge and training about medical cannabis (20, 29–31). In previous survey studies, approximately 60% of physicians noted that they did not receive any education regarding medical cannabis (31, 32). In a study among Israeli primary care physicians, 63% of respondents endorsed having little knowledge, and 75% noted a need for greater education regarding medical cannabis (31). In another study, only 51% of clinicians (including pharmacists, nurse practitioners, and physician assistants) reported completing any formal training on medical cannabis (33). In a national survey of US medical school deans, residents, and fellows from 145 schools, 66.7% of deans reported that their graduates were not educated about medical cannabis, 84.9% of residents noted receiving no education on medical cannabis in medical school or residency, and only 9% of medical schools documented medical cannabis education in the AAMC Curriculum Inventory database (28).

In this study, when assessing the overall willingness to recommend medical cannabis for treating a variety of severe disorders, a large amount of heterogeneity was noted. Of the 20 clinical vignettes/disorders presented, many responders were overall willing to recommend medical cannabis for chemotherapy-induced effects, chronic spasticity associated with relapsing multiple sclerosis (MS)/amyotrophic lateral sclerosis (ALS), and HIV-induced cachexia.

Current evidence-based recommendations by the medical community do not recommend the routine use of cannabis for neurologic and psychiatric disorders. The American Academy of Neurology (AAN) published a ‘systematic review’ with the conclusion that oral cannabis extract and THC ‘are probably effective’ in reducing patient-centered measures and spasticity-related pain. The evidence was noted to be insufficient for tremors, urinary dysfunction, Parkinson’s dyskinesia, and Tourette syndrome (34, 35). The American Psychiatric Association (APA) has issued an official action in their ‘position statement in Opposition to Cannabis as medicine’, which concluded that “there is no current scientific evidence that cannabis is in any way beneficial for the treatment of any psychiatric disorder. In contrast, current evidence supports, a strong association of cannabis use with the onset of psychiatric disorders” (36).

At odds with the above-cited guidelines, approximately 40% of responders suggested a willingness to recommend medical cannabis for conditions for which guidelines prohibit use; 20–30% of responders endorsed willingness to use medical cannabis in post-traumatic stress disorder (PTSD), autism, and tic disorder. Conversely, less than half of the responders correctly identified the probability of addiction after daily use of cannabis. Taken together, the findings consistently support the notion that there is a severe gap in domain-specific knowledge related to cannabis among physicians. The inconsistency in individual physician practice results in the underutilization of medical cannabis where a reasonable evidence base exists. While the AAN guidelines overall endorse using medical cannabis for severe spasticity in MS/ALS, only one in two physicians expressed a willingness to act in accordance with the medical society’s guidelines. In the study among Israeli primary care physicians, respondents were also found to be less likely to initiate medical cannabis but were willing to renew a prescription for medical conditions, excluding PTSD, chronic pain, and fibromyalgia (31).

We also examined how physicians’ personal beliefs about medical cannabis impact clinical practice and their willingness to recommend medical cannabis in this cohort (37). When queried about their individual beliefs regarding cannabis and its clinical value, just over half of the physicians endorsed a willingness to use medical cannabis for themselves if diagnosed with a qualifying condition. Physicians with a personal belief in favor of the utility of cannabis were more likely to recommend cannabis for other medical conditions (Supplementary Table 1). We have previously shown that personal belief regarding the utility of medical cannabis was significantly associated with physicians’ willingness to recommend medical cannabis, even after controlling for knowledge related to medical cannabis (37). This is consistent with a study among primary care physicians where willingness to recommend medical cannabis increased significantly for respondents who believed that medical cannabis was effective (31). In the absence of adequately powered randomized controlled trials, this belief regarding the utility of medical cannabis may be driven by anecdotal reports or misinformation in the lay media.

Taken together, the findings suggest that medical decisions related to medical cannabis are guided by clinical experience and personal beliefs amid insufficient evidence. Perhaps unsurprisingly, this situation prompts the need for individual physicians to implement heuristic and non-scientific-based reasoning to arrive at the decision to recommend or use medical cannabis. The results suggest that there are other factors impacting a decision to withhold or propose medical cannabis as a course of treatment. Concordantly, the variance accounted for this predictive model was approximately 10% for the aggregate of clinical cases (37).

The results of this study need to be viewed in the context of its limitations. The study was observational in nature and based on responses from physicians in countries with various legal statuses of medical cannabis. Physicians’ prior experience with medical cannabis was not systematically assessed. Additionally, the sample size was small for many individual countries. This limits the representativeness of the sample and the ability to make country-specific inferences. These limitations notwithstanding, the study points to the importance of addressing the gap in knowledge and medical training about medical cannabis, and the need for guidelines to inform physician practices related to medical cannabis.

## Data Availability

The original contributions presented in the study are included in the article/Supplementary material, further inquiries can be directed to the corresponding author.
