# Supplementary material for: International perspectives on physician knowledge, attitudes, and practices related to medical cannabis
Source: Front Public Health. 2025 Feb 20;13:1463871. doi: 10.3389/fpubh.2025.1463871 (PMC11882600; doi:10.3389/fpubh.2025.1463871)
Supplement: Supplementary file 1 [file Table_1.docx]

**Supplementary Table 1 : Difference in willingness to recommend based on personal belief about the utility of medical cannabis**

|  | | | |  |  |
| --- | --- | --- | --- | --- | --- |
| Case vignette | Belief about utility of medical cannabis | Mean % | SD | t | Significance |
| 35 year old male veteran with post-traumatic stress disorder (PTSD) | Y | 34.88 | 29.61 | -4.19 | <0.001 |
|  | N | 17.14 | 24.07 |  |  |
| 70 year old male with diabetes mellitus and refractory neuropathic pain | Y | 56.31 | 31.91 | -2.52 | 0.013 |
|  | N | 43.93 | 32.36 |  |  |
| 29 year old male with HIV/AIDS and poor appetite/cachexia | Y | 53.86 | 32.71 | -2.33 | 0.021 |
|  | N | 41.34 | 34.83 |  |  |
| 44 year old female with opioid abuse and anxiety attacks. | Y | 26.63 | 27.62 | -2.59 | 0.011 |
|  | N | 15.28 | 24.55 |  |  |
| 55 year old female with acute myeloid  leukemia (AML) and chemotherapy-induced nausea | Y | 73.27 | 30.97 | -3.25 | 0.001 |
|  | N | 56.5 | 35.42 |  |  |
| 54 year old male with severe rheumatoid arthritis | Y | 51.55 | 31.16 | -2.26 | 0.024 |
|  | N | 40.45 | 30.55 |  |  |
| 15 year old male with sickle cell disease | Y | 26.02 | 26.95 | -1.99 | 0.048 |
|  | N | 16.52 | 24.04 |  |  |
| 8 year old female with Autism and self- injurious behavior | Y | 29.14 | 30.29 | -4.02 | 0.0001 |
|  | N | 10.98 | 21.21 |  |  |
| 67 year old male with severe Parkinson disease | Y | 43.73 | 30.38 | -2.04 | 0.044 |
|  | N | 32.71 | 34.47 |  |  |
| 32 year old female with insomnia and no significant medical or psychiatric history | Y | 21.51 | 27.60 | -3.35 | 0.001 |
|  | N | 8.02 | 19.16 |  |  |
| 85 year old male with Lewy body dementia  and severe agitation (intolerant to antipsychotics) | Y | 51.12 | 36.07 | -4.36 | 0.0001 |
|  | N | 27.49 | 31.48 |  |  |
| 13 year old female with Tourette’s syndrome and uncontrolled secondary tics | Y | 33.69 | 31.67 | -4.70 | 0.0001 |
|  | N | 12.64 | 18.73 |  |  |
| 24 year old male with uncontrolled epilepsy, on three anti-epileptic drugs | Y | 44.06 | 34.89 | -2.51 | 0.012 |
|  | N | 29.6 | 32.35 |  |  |
| 23 year old female with amyotropic lateral sclerosis (ALS) severe remitting relapsing  type | Y | 54.22 | 35.28 | -3.54 | 0.001 |
|  | N | 34.2 | 32.36 |  |  |
|  | Y | 24.17 | 28.72 | -2.64 | 0.009 |

| 56 year old male, with alcohol abuse, hepatitis C and recent cirrhosis diagnosis | N | 12.46 | 24.53 |  |  |
| --- | --- | --- | --- | --- | --- |
| 36 year old male with amyotropic lateral sclerosis (ALS) and severe spasticity | Y | 63.29 | 34.81 | -4.64 | 0.0001 |
|  | N | 36.11 | 35.25 |  |  |
| 28 year old male with severe psoriasis without arthritis. | Y | 29.88 | 33.07 | -2.85 | 0.005 |
|  | N | 16.03 | 22.67 |  |  |
| 48 year old female with refractory glaucoma | Y | 41.6 | 36.39 | -2.02 | 0.045 |
|  | N | 29.37 | 32.40 |  |  |
| 31 year old male with multiple sclerosis and severe spasticity | Y | 62.27 | 33.02 | -4.96 | 0.001 |
|  | N | 34.1 | 33.66 |  |  |
| 36 year old male with chronic/refractory Crohn’s disease | Y | 47.9 | 31.05 | -4.64 | 0.001 |
|  | N | 23.83 | 27.18 |  |  |
