# Supplementary material for: International perspectives on physician knowledge, attitudes, and practices related to medical cannabis
Source: Front Public Health. 2025 Feb 20;13:1463871. doi: 10.3389/fpubh.2025.1463871 (PMC11882600; doi:10.3389/fpubh.2025.1463871)
Supplement: Supplementary file 2 [file Data_Sheet_1.pdf]

# International Medical Cannabis Survey

Q1

Choose the region of the world that you live in:

Choose country

If you are in USA, choose state

Q2

Please enter your National Provider Identification (NPI) number (if you are in USA) OR the national Medical Council registration number of your country to authenticate that you are a physician. By entering your NPI or Medical council registration number you are also indicating your consent to participate in this survey.

☐

Q3

Enter the words or numbers that you see in the textbo below:

Q4

If you are unable to access the survey, or want to access this survey in another language please send an email to: rajiv.radhakrishnan@yale.edu

Q5

Please tell us a little about yourself

| | |
|---|---|
| Year of birth | ☐ |
| Gender (M/F) | ☐ |
| What state/states/region do you practice in | ☐ |
| Year of graduating residency/ completing medical training (or expected year) | ☐ |
| Is medical use of marijuana legalized in the state/region that you practice in? (Yes/ No/ Don't know) | ☐ |

Q6

Please tell us about your training and current practice.

Specialty of residency training

Sub-specialty training

Practice Setting

Q7

How would you best characterize your practice?

Primarily In-patient

Primarily Out-patient

Q8

Approximately what percentage of patients you see belong to the following categories?

| | 0 | 10 | 20 | 30 | 40 | 50 | 60 | 70 | 80 | 90 | 100 |
|---|---|---|---|---|---|---|---|---|---|---|---|
| Children and adolescents | | | | | | | | | | | |
| Adults | | | | | | | | | | | |
| Geriatrics | | | | | | | | | | | |

Q9

Approximately what percentage of patients that you see in your practice have one or more the following diagnosis?

| | 0 | 10 | 20 | 30 | 40 | 50 | 60 | 70 | 80 | 90 | 100 |
|---|---|---|---|---|---|---|---|---|---|---|---|
| Pain syndromes | | | | | | | | | | | |
| Terminal Cancer/ Terminal illness | | | | | | | | | | | |
| Epilepsy | | | | | | | | | | | |
| Multiple sclerosis | | | | | | | | | | | |
| Alzheimer's disease | | | | | | | | | | | |
| Tourette syndrome | | | | | | | | | | | |
| Substance dependence/ Addiction | | | | | | | | | | | |
| Depression, mania and other mood disorders | | | | | | | | | | | |
| Schizophrenia | | | | | | | | | | | |
| Post-Traumatic Stress | | | | | | | | | | | |

| Disorder |
| --- |
| Sickle cell disease |
| Crohn's disease |
| Psoriasis |
| Hepatitis C |
| Arthritis |

Q10

Please indicate your proficiency in treating the following conditions (2 stars= novice; 3 stars= moderate proficiency; 5 stars= expert)

Pain syndromes

Terminal illness/ cancer

Epilepsy

Multiple Sclerosis

Alzheimer's Disease

Tourette's syndrome

Substance abuse/addiction

Depression, mania and other mood disorders

Schizophrenia

Post-traumatic Stress Disorder

Sickle-cell disease

Crohn's disease

Psoriasis

Hepatitis C

Arthritis

Q11

If you have received requests from patients for "medical-marijuana"

| | |
|---|---|
| How many requests to you get on average in a month? | ☐ |
| What percent of the patients you see request "medical marijuana" | ☐ |
| The 1st most common reason for request of "medical mariijuana" is: | ☐ |
| The 2nd most common reason for request of "medical marijuana" is: | ☐ |
| The 3rd most common reason for request of "medical marijuana" is: | ☐ |

Q12

What has your experience with prescribing "medical marijuana" been?

I frequently prescribe/authorize

"medical marijuana" I refer patients

who need "medical marijuana"

I've never prescribed/authorized "medical marijuana" but I'm open to the idea

I've never prescribed/authorized "medical marijuana" and I never will

I don’t know enough about "medical marijuana" to make a decision

Q13

Recognizing that the decision to prescribe a medication is highly dependent on the patient and their specific and unique circumstances, how willing are you to prescribe/authorize/recommend "medical marijuana" in the following case vignettes? In all of these cases, the patient has approached you asking for a medical marijuana recommendation. Choose a percentage between (0%- Definitely no, 50%- Neither likely nor unlikely, 100%- Definitely yes; Skip question - not my specialty).

| | 0 | 10 | 20 | 30 | 40 | 50 | 60 | 70 | 80 | 90 | 100 |
|---|---|---|---|---|---|---|---|---|---|---|---|
| 1. 35y/o male veteran with post-traumatic stress disorder (PTSD). | | | | | | | | | | | |
| 2. 70-year-old man with diabetes and chronic neuropathic pain refractory to gabapentin. | | | | | | | | | | | |
| 3. 29y/o woman with HIV/AIDS complaining of low appetite, significant weight loss and cachexia. | | | | | | | | | | | |
| 4. 44 year-old woman with opioid abuse (prescribed for non-specific pain) presenting with “anxiety attacks.” | | | | | | | | | | | |

5. 55-year old woman with severe nausea/vomiting undergoing chemotherapy for acute myelogenous leukemia.

6. 54-year-old man with severe rheumatoid arthritis

Q14

Recognizing that the decision to prescribe a medication is highly dependent on the patient and their specific and unique circumstances, how willing are you to prescribe/authorize/recommend "medical marijuana" in the following case vignettes? In all of these cases, the patient has approached you asking for a medical marijuana recommendation. Choose a percentage between (0%- Definitely no, 50%- Neither likely nor unlikely, 100%- Definitely yes; Skip question - not my specialty).

| | 0 | 10 | 20 | 30 | 40 | 50 | 60 | 70 | 80 | 90 | 100 |
|---|---|---|---|---|---|---|---|---|---|---|---|
| 7. 15 year-old male with severe Sickle cell disease. | | | | | | | | | | | |
| 8. 8-year-old girl with severe autism and behavioral problems, self- injurious behavior and agitation. | | | | | | | | | | | |
| 9. 67-year-old man with history of moderate-severe Parkinson's disease. | | | | | | | | | | | |
| 10. 32-year-old woman with no medical history presenting with insomnia. | | | | | | | | | | | |

11. 85 year old man with Alzheimer's disease/ Lewy body dementia with extreme sensitivity to antipsychotics who has uncontrolled episodic agitation.

12. 13 year old girl with severe uncontrolled tics secondary to Tourette's disease.

13. 24 year old male with epilepsy not controlled with 3 anti-epileptics.

Q15

Recognizing that the decision to prescribe a medication is highly dependent on the patient and their specific and unique circumstances, how willing are you to prescribe/authorize/recommend "medical marijuana" in the following case vignettes? In all of these cases, the patient has approached you asking for a medical marijuana recommendation. Choose a percentage between (0%- Definitely no, 50%- Neither likely nor unlikely, 100%- Definitely yes; Skip question - not my specialty).

| | 0 | 10 | 20 | 30 | 40 | 50 | 60 | 70 | 80 | 90 | 100 |
|---|---|---|---|---|---|---|---|---|---|---|---|
| 14. 23 year old female with severe remitting-relapsing Multiple Sclerosis with recent onset of optic neuritis | | | | | | | | | | | |
| 15. 56 year old man with history of alcohol abuse, hepatitis C, recently diagnosed with cirrhosis | | | | | | | | | | | |
| 16. 36 year old man with Amyotropic Lateral Sclerosis with severe spasticity | | | | | | | | | | | |
| 17. 28 year old man with severe psoriasis without psoriatic arthritis | | | | | | | | | | | |

18. 48 year old female with severe, refractory glaucoma

19. 31 year old man with severe spasticity secondary to Multiple Sclerosis

20.36 year old male with chronic, refractory Crohn's Disease.

Q16

Approximately what percentage of patients that you see in your practice have the following diagnosis?

| | 0 | 10 | 20 | 30 | 40 | 50 | 60 | 70 | 80 | 90 | 100 |
|---|---|---|---|---|---|---|---|---|---|---|---|
| Cannabis-induced psychosis | | | | | | | | | | | |
| Synthetic cannabinoid-induced psychosis (Spice/K2) | | | | | | | | | | | |
| Bath-salt (cathinone)-induced psychosis | | | | | | | | | | | |
| Alcohol-induced psychosis | | | | | | | | | | | |
| Exacerbation of pre-existing psychiatric disorder due to cannabis use | | | | | | | | | | | |

Exacerbati
on of
medical
disorder due
to cannabis
use

Q17

Approximately what percent of people who try marijuana (just once) develop addiction or use disorder?

<1%

5%

10%

>20%

Q18

Without using an internet search, can you name the qualifying conditions for "medical marijuana" according to the laws in your State (choose all that apply)

Post-traumatic Stress Disorder

HIV/

AIDS

Glauco

ma

Cancer

Neuropathic

pain Chronic

low back pain

Epilepsy

Schizophrenia

Non-specific nausea/vomiting

Cluster headache

Amyotropic Lateral Sclerosis (ALS or Lou

Gehrig's disease) Crohn's disease

Juvenile Rheumatoid Arthritis

Alzheimer's

disease

Multiple

Sclerosis

Hepatitis C

Psoriasis

Fabry's

disease

Sickle cell

disease

Severe spasticity secondary to neurological cause

None of the above/ Does not apply to my country/region

Q19

Approximately what percent of people who use marijuana daily are or will become addicted?

<1%

10-15%

25-50%

>50%

Q20

In your opinion, are there reasons to prescribe medical marijuana rather than dronabinol (delta-9-tetrahydrocannabinol), which is approved by Food and Drug Administration, European Medicines Agency or equivalent national regulatory agency? (If yes, list up to 5 reasons, one in each line)

Q21

Do you believe there is a link between cannabis use and psychosis?

Yes, I've seen cases that exemplify the association

Yes, I believe the research studies

No, the link between cannabis use and psychosis is an artifact

No, I've never seen a cases of psychosis among my patients who smoke a lot of cannabis

No, I don't believe the research studies

Q22

Hypothetically, if you had a condition that qualified for "medical marijuana" would you opt to get a prescription for yourself?

Yes

No
